# Basophils from allergy to cancer

**DOI:** 10.3389/fimmu.2022.1056838

**Published:** 2022-12-12

**Authors:** Remo Poto, Adriana Rosa Gambardella, Gianni Marone, John T. Schroeder, Fabrizio Mattei, Giovanna Schiavoni, Gilda Varricchi

**Affiliations:** ^1^ Department of Translational Medical Sciences, University of Naples Federico II, Naples, Italy; ^2^ Department of Oncology and Molecular Medicine, Istituto Superiore di Sanità, Rome, Italy; ^3^ World Allergy Organization (WAO), Center of Excellence (CoE), Naples, Italy; ^4^ Center for Basic and Clinical Immunology Research (CISI), University of Naples Federico II, Naples, Italy; ^5^ Institute of Experimental Endocrinology and Oncology “G. Salvatore”, National Research Council (CNR), Naples, Italy; ^6^ Department of Medicine, Division of Allergy and Clinical Immunology, Johns Hopkins Asthma and Allergy Center, Johns Hopkins University, Baltimore, MD, United States

**Keywords:** allergy, angiogenesis, angiopoietins, basophil, cancer, cysteinyl leukotrienes, cytokines, vascular endothelial growth factors

## Abstract

Human basophils, first identified over 140 years ago, account for just 0.5-1% of circulating leukocytes. While this scarcity long hampered basophil studies, innovations during the past 30 years, beginning with their isolation and more recently in the development of mouse models, have markedly advanced our understanding of these cells. Although dissimilarities between human and mouse basophils persist, the overall findings highlight the growing importance of these cells in health and disease. Indeed, studies continue to support basophils as key participants in IgE-mediated reactions, where they infiltrate inflammatory lesions, release pro-inflammatory mediators (histamine, leukotriene C_4_: LTC_4_) and regulatory cytokines (IL-4, IL-13) central to the pathogenesis of allergic diseases. Studies now report basophils infiltrating various human cancers where they play diverse roles, either promoting or hampering tumorigenesis. Likewise, this activity bears remarkable similarity to the mounting evidence that basophils facilitate wound healing. In fact, both activities appear linked to the capacity of basophils to secrete IL-4/IL-13, with these cytokines polarizing macrophages toward the M2 phenotype. Basophils also secrete several angiogenic factors (vascular endothelial growth factor: VEGF-A, amphiregulin) consistent with these activities. In this review, we feature these newfound properties with the goal of unraveling the increasing importance of basophils in these diverse pathobiological processes.

## Introduction

Paul Ehrlich discovered, over 140 years ago, peripheral blood basophils and tissue mast cells using novel hematological techniques that combined the use of alkaline dyes and conventional light microscopy (1, 2). Unlike mast cells, which are only found as mature cells in tissues, basophils represent just 0.5-1% of all leukocytes in the bone marrow and peripheral blood (3, 4). Basophils and mast cells are long recognized as being morphologically similar in appearance and for sharing several unique features (5, 6). For example, they are the only two cells that express the full tetrameric (αβγ2) form of the high-affinity receptor for IgE (FcϵRI). They both also uniquely store histamine in cytoplasmic granules (7), releasing it and other proinflammatory mediators (e.g., cysteinyl leukotrienes) when appropriately activated (5, 8). In fact, these shared characteristics continue to cause misperceptions, leading some to believe that basophils and mast cells are one and the same. However, compelling evidence over the last decades now supports that human basophils possess morphological, immunological, biochemical, and pharmacological characteristics quite different from those of human mast cells (5–7, 9).

Until recently, there was some dispute as to whether mice have basophils. However, the work of Ann M. Dvorak using electron microscopy, clearly identified basophils in mice as a rare population of bone marrow cells, with some ultrastructural characteristics like those observed in human basophils (7, 10, 11). As discussed below, there remains considerable debate as to whether mouse basophils are truly representative of human cells, particularly with regard to function (11–17). Of course, much of this debate often defaults to issues pertaining to the disparities expected with *in vitro vs*. *in vivo* experiments (18). Nonetheless, the one newest function perhaps most shared by basophils from both species is their capacity to secrete large quantities of IL-4, even though debate persists about the stimuli most responsible for this response.

Many fundamentals of basophil biology have been extensively reviewed elsewhere, especially regarding their role in allergic diseases (5, 19–24). In this review, we briefly touch on this research field but will additionally focus on the concept of basophils participating in tumorigenesis and wound-healing and how these processes are seemingly linked and driven by the capacity of these cells to secrete IL-4, IL-13, angiogenic factors and pro-fibrotic cytokines.

### Basophil development

Basophils originate from stem cell progenitors in the bone marrow (25–27). Both in humans and mice, IL-3 is the most important growth factor for basophil development (12, 17, 28, 29). In fact, basophils from both species can be developed *in vitro by* simply culturing bone marrow cells (or CD34^+^ precursors in humans) in the presence of IL-3 for 10-14 days (12, 30–32).

While IL-3 is clearly most important for basophil development from precursors, other growth factors are reported to facilitate expansion/function. For example, the FMS-like tyrosine kinase 3 ligand (Flt3L) has been combined with IL-3 to expand the number of culture-derived basophils (33). Siracusa and co-workers reported that mouse basophils can be generated by thymic stromal lymphopoietin (TSLP) through the engagement of the heterodimeric TSLP receptor (TSLPR/IL-7Rα) (34). These authors demonstrated that IL-3 and TSLP induced the differentiation of two types of murine basophils displaying different gene expression and functions (35). In humans, it has been suggested that about 10% of basophils from asthmatics express the TSLP receptor and release histamine and cytokines in response to TSLP (15). In contrast, more recent studies have shown that human basophils do not express the IL-7Rα subunit of the heterodimeric TSLP receptor (14) and do not respond to *in vitro* TSLP stimulation (12, 14, 16). By contrast, TSLP induces the release of IL-4, IL-13, CXCL1, and CXCL2 from mouse basophils (12).

### Heterogeneity of basophils: In species and tissue *versus* peripheral blood

Human and mouse basophils express FcεRI (36, 37) and will up-regulate the degranulation markers, CD63 (38–40) and CD203c when activated appropriately (9, 39, 41–43). Basophils from humans and mice express the IL-3 (IL-3Rα/CD123) (34, 44), GM-CSF (CD116) (45, 46), and IL-33 (ST2/IL1RL1) receptors (47–50). The heterodimeric TSLP receptor, TSLPR/IL-7Rα, is expressed by mouse basophils (12, 34), but the presence of this receptor on basophils from allergic donors and healthy subjects remains controversial (12, 14–16). Human basophils reportedly express receptors for IL-5 (CD125) (51) and for Nerve Growth Factor (NGF) (tropomyosin receptor kinase A: TrkA) (52–54). Both human and mouse basophils display a variety of chemokine receptors (5, 55–60). The IgG receptors FcγRIIA, FcγRIIB, and small amounts of FcγRIIIB are expressed by human basophils, whereas mouse basophils express FcγRIIB and FcγRIIIA (61, 62).

Preformed mediators, such as histamine (≃ 1 pg/cell), basogranulin (63, 64) and very low concentrations of tryptase (65) are present in human basophils. Human (66) and mouse basophils release granzyme B (67), that reportedly exerts cytotoxic effects on tumor cells (68, 69). Basophils from both species can synthesize cysteinyl leukotriene C_4_ (LTC_4_) through the 5-lipoxygenase pathway (70). Mouse basophils additionally produce prostaglandin D_2_ (PGD_2_) and prostaglandin E_2_ (PGE_2_) through the cyclooxygenase pathway (71, 72). Human basophils do not synthesize detectable levels of PGD_2_ or other mediators requiring cyclooxygenase activity (12, 73).

Substantial evidence now shows that human (12, 24, 74–82) and mouse (12, 47, 80) basophils secrete IL-4. Both human (12, 75, 76, 78, 81–85) and mouse basophils (12, 47) also generate and release IL-13, yet the evidence for this response is far more prevalent in the former species. Mouse basophils can release IL-6 (47, 86, 87) and TNF-α (47, 86). Two reports indicate that these cytokines are secreted from human basophils (88, 89), even though they do not appear to be products commonly released by these cells. Human and mouse basophils release granzyme B (66, 67) that exerts a cytotoxic effect on tumor cells.

Human basophils secrete several angiogenic factors such as vascular endothelial growth factor-A (VEGF-A) (64), angiopoietin-1 (ANGPT1) (90), hepatocyte growth factor (HGF) (47, 91), and amphiregulin (AREG) (92–94). Mouse (47) and human basophils (91) express *Hgf* and release, under certain conditions, AREG (94) and VEGF-A (Gambardella et al., unpublished).

The life-span of circulating basophils is relatively short (≃ 2.5 days in mice) (95) and therefore newly generated basophils are constantly supplied from the bone marrow to the blood (25). Basophils physiologically circulate in peripheral blood and migrate within tissues mainly during certain types of inflammation in mice (86, 95–98) and humans (99–104). Basophils, present during mouse lung development, exhibit a phenotype different from circulating blood basophils (47). In the lung, specific gene signature of lung-resident basophils is modulated by IL-33 and GM-CSF (47). These cells play a prominent role in the development and polarization toward the M2 state of alveolar macrophages, raising the possibility that in tumors associated with M2 macrophages (105–107), basophils contribute the polarization of tumor-associated macrophages.

Basophils derived from murine bone marrow cells are often used as a model system for studies of the immunological functions of these cells (86, 97, 108–111). It should be pointed out that these cells, developed by murine bone marrow cells in the presence of IL-3, have an activated phenotype (82, 112). Recently, Pellefigues et al. carefully demonstrated functional heterogeneity between naïve murine basophils obtained from spleen and bone marrow-derived basophils (108). In humans, functional heterogeneity of peripheral blood basophils has been demonstrated by applying mass cytometry (CyTOF) to simultaneously assess several proteins and functions of basophils (113).

### Angiogenic factors released by basophils

Angiogenesis occurs physiologically during embryonic development, pathologically in inflammation and cancer (114, 115). Both cancer and immune cells (116, 117) produce several proangiogenic factors (118, 119). The vascular endothelial growth factor (VEGF) family includes VEGF-A, VEGF-B, VEGF-C, and VEGF-D. VEGFs activate specific receptors (VEGFR1, VEGFR2, and VEGFR3) on blood endothelial cells (BECs). VEGF/VEGFR axis plays pivotal roles in tumor and inflammatory angiogenesis (118). VEGF-A is released by human basophils (64). All members of the VEGF family are chemotactic for human basophils through the engagement of VEGFR2 on their surface (64, 120). Therefore, VEGFs released by cancer cells and immune cells in the tumor microenvironment (TME) (118, 120–123) can favor basophil infiltration in TME.

Angiopoietins (ANGPTs) are other players of inflammatory and tumor angiogenesis (124, 125). ANGPT1, released by perivascular mural cells, binds to the Tie2 receptor on endothelial cells and promotes endothelial stabilization (126). ANGPT2, secreted by activated endothelial cells, induces vascular permeability (127). ANGPT1 and ANGPT2 mRNAs are expressed by human basophils (90), and their activation induces ANGPT1 release. Mouse lung-resident basophils express mRNA for HGF, a potent angiogenic factor (47, 91, 128).

Cysteinyl leukotrienes (cys-LTs) are powerful proinflammatory mediators (129). The cys-LTs include leukotriene C_4_ (LTC_4_), the main lipid mediator synthesized by human and mouse basophils (54, 70). ɤ-glutamyl transpeptidases metabolize LTC_4_ to LTD_4_ and to LTE_4_ by the membrane-bound enzymes (129). Cys-LTs are potent agonists of three different receptors (CysLTRs) CysLT_1_R, CysLT_2_R, and CysLT_3_R (130–132). LTC_4_ and LTD_4_ induced the formation of angiogenesis (133). The angiogenic properties of LTC_4_ and LTD_4_ were mediated *in vivo* by the activation of CysLT_2_R on BECs. In mouse models, pharmacologic antagonism of CysLT_2_R inhibited tumor growth and metastasis formation (133). These results illustrate the relevance of cys-LTs as non-canonical angiogenic factors in cancer. Moreover, these findings suggest that CysLT_2_R might be a target in cancer (133). LTC_4_ is released by activated human (70, 134) and mouse (54) basophils and future studies should investigate whether basophil-derived LTC_4_ might contribute to angiogenesis in human cancer.

### Formation of extracellular DNA traps by basophils

Activated neutrophils (135–137), eosinophils (138, 139), mast cells (140–143), macrophages (144–148), and basophils (149, 150) can release extracellular traps (ETs), which are DNA structures decorated with a variety of proteins [e.g., myeloperoxidase and elastase) (151), lactoferrin and pentraxin 3) (151, 152), and matrix metalloproteinase 9) (151)]. ETs released by human neutrophils (neutrophils extracellular traps: NETs) were initially characterized by their antibacterial activity (138, 151, 153, 154). Increasing evidences demonstrate that ETs, particularly NETs, play a role in asthma (137) and in fundamental aspects of tumorigenesis (155). NETs favor the formation of metastasis in mice and in humans (156–159) and awaken dormant cancer cells (160). An increase of NET release occurs when neutrophils from myeloproliferative neoplasms are associated with *JAK2* ^V617F^ mutations and mice with knock-in of *JAK2* ^V617F^ (161). We have provided evidence that anaplastic thyroid cancer cells can induce NET formation (162). Collectively, these findings demonstrate that NETs can promote tumor growth and metastasis formation. Basophils from humans and mouse can release extracellular DNA traps (BETs) *in vitro* and *in vivo* (149, 150, 163). The translational relevance of these findings should be explored in experimental models and human cancers.

### Basophils in allergic disorders

Basophils play a major role in a variety of allergic disorders (8, 164–166). Anaphylaxis is a rapid-onset, potentially life-threatening allergic reaction caused by the release of vasoactive mediators from mast cells and basophils after allergen exposure (167). Mouse models of anaphylaxis suggest that basophils play a major role in the IgG-, but not IgE-mediated anaphylaxis (168). In these studies, the depletion of basophils by anti-CD200R3 mAb inhibited IgG-mediated anaphylaxis, whereas it had minor effect on IgE-mediated anaphylaxis. By contrast, mast cells are central for IgE-mediated mouse models of anaphylaxis (168, 169).

Several lines of indirect evidence suggest that basophils participate in human anaphylaxis (24). For example, the number of circulating basophils was significantly lower in subjects undergoing anaphylactic reactions compared to healthy controls (170). Peanut-induced allergic reactions also resulted in a significant decrease in circulating basophil counts and an increase in CCL2 levels compared with those in pre-challenge samples.

While there is a plethora of information from murine models regarding the role of basophils in allergic/asthma-like inflammation, the involvement of basophils in human asthma again derives mainly from indirect evidence (164). Most compelling, basophils have been found in the airways of asthmatics (171, 172), in post-mortem cases of fatal asthma (173) and after antigen challenge of airway mucosa (174). Basophil releasability (i.e., the ability of a basophil to release a given percentage of histamine in response to a given immunological stimulus) is long reported to be increased in asthma and more recently subject to circadian changes (175). Moreover, allergen-induced asthmatic responses are accompanied by infiltration of basophils expressing IL-4 mRNA (103). The *in vitro* secretion of both IL-4 and IL-13 has been shown to track with the basophil-enriched fractions of cells recovered after infiltrating the lung following segmental allergen challenge (176, 177). Moreover, these so-called basophil cytokine responses also correlated with the frequency of eosinophils recovered from the lung. Thus, basophils might represent an important source of Th2-like cytokines (IL-4 and IL-13) in the lung microenvironment, particularly that associated with human allergic disease.

Brooks and collaborators reported that basophils are increased in the sputum of patients with eosinophilic asthma compared to those with non-eosinophilic asthma (178). In asthmatics, basophils were positively correlated with sputum eosinophils and inversely with sputum neutrophils, but not with FEV_1_, FEV_1_/FVC or bronchodilator reversibility. Sputum basophils positively correlated with sputum eosinophils (179). In comparison with blood basophils, sputum basophils have a higher expression of activation markers (e.g., CD203c) (179). These findings indicate that basophils may be involved in eosinophilic asthma and that sputum basophil assessment could be a useful additional indicator of “Th2-high” asthma. Basophil counts in peripheral blood during childhood asthma are associated with exacerbations (180). The proportion of degranulated basophils can also be associated with recurrent exacerbations.

Hill et al. reported that omalizumab, a mAb that targets IgE and neutralizes it from binding to FcεRIα on basophils, reduces blood basophil frequencies in asthmatic children (181). Furthermore, treatment of severe asthma patients with benralizumab, a mAb against IL-5Rα, markedly decreased the number of both eosinophils and basophils (182–184). These findings suggest that benralizumab may have a positive effect on severe asthmatics by reducing not only eosinophils but also basophils.

A number of mouse studies indicate that basophils are involved in the development of asthma-like pathology. In an ovalbumin-induced asthma model, basophils recruited to the lungs, amplify the Th2 cell differentiation (185). In a papain-induced asthma model, basophil-derived IL-4 induces the IL-5 and CCL11 expression in ILC2 cells, causing eosinophil infiltration (68). Indeed, in a model of IgE-dependent dermatitis, the production of IL-4 from basophils was shown to directly condition endothelium for increased VCAM-1 expression, which facilitated the *in vivo* entry of eosinophils into lesion sites (186). This mechanistic observation may help elucidate the eosinophil/basophil IL-4 associations commonly seen in human disease.

Chronic spontaneous urticaria (CSU) is a common skin disease, characterized by spontaneous appearance of wheals, angioedema or both, for more than 6 weeks due to known or unknown causes (187, 188). A role for basophils in the pathophysiology of CSU is suggested by a number of findings (189, 190). CSU subjects have been shown to have significant increases in the numbers of intradermal basophils compared with non-atopic control subjects (191). Basopenia has long been reported in patients with CSU (192) and more recently postulated as the result of basophil migration from the circulation into the skin (104, 191, 193). The degree of basopenia often correlates with disease severity (194) and improves during times of remission (195). CSU subjects exhibit enhanced expression of the activation markers CD63 and CD69 on basophils compared to non-allergic subjects (196).

Rauber et al. identified three distinct immunologic phenotypes of CSU (197). One group of patients’ basophils reacted to FcϵRI stimulation, whereas the others had anti-FcϵRI nonreactive basophils. Among the latter, it was found a subgroup with basopenia. This subgroup had augmented serum-induced basophil activation, increased levels of autoantibodies against thyroid peroxidase, and worse quality of life. These phenotypes were associated with different clinical characteristics, pointing to basophils as important players in CSU pathophysiology (197). Oda et al. demonstrated that basophils from CSU patients had higher FcεRI expression compared to healthy controls. The proportion of CD203c^high^ basophils after anti-IgE or anti-FcεRI stimulation was lower in CSU patients compared to controls and characteristics of more severe patients (198).

Omalizumab is a mAb anti-IgE often used in treating severe allergic asthma (199, 200). More recently, it has also proved highly effective in patients with CSU (201). Surprisingly, this treatment, regardless of the disease being treated, is associated with increased expression of Syk, which is often also manifested by basophils showing greater histamine release *in vitro* when undergoing IgE/FcεRIα-dependent stimulation. This enhanced responsiveness is seen even through cell-surface FcεRIα/IgE levels are reduced with this treatment (196, 202). These observations have since prompted the same group of authors to suggest that Syk expression and IgE-mediated histamine release in basophils could function as biomarkers for predicting the clinical efficacy of omalizumab in patients receiving this therapy (203).

In following CSU subjects treated with omalizumab, MacGlashan and collaborators have also identified three basophil phenotypes in CSU patients: 1) subjects with basopenia, 2) normal basophil numbers with normal IgE-mediated histamine release, and 3) normal basophil numbers with poor histamine release. Basopenia was associated with the presence of autoantibodies to unoccupied FcϵRI and basophil numbers did not change during omalizumab treatment. Omalizumab resulted in similar kinetics for decreases in surface FcϵRI and IgE in all three groups of CSU patients (204).

Atopic dermatitis is a common inflammatory skin disorder characterized by chronic eczema and severe itching (205). Th2 cells mediate inflammation in atopic dermatitis with the release of IL-4 and IL-13, which contribute to clinical manifestations (206, 207). Keratinocyte-derived alarmins, such as IL-33, TSLP, and IL-25 (IL-17E) that elicit Th2 cytokines responses by activating group-2 innate lymphoid cells (ILC2s) play an upstream pathogenic role in atopic dermatitis (208, 209). Recent evidence indicates that LTC_4_ also plays a role in mouse models of atopic dermatitis (210). IgG autoantibodies against IgE from atopic dermatitis can induce the release of IL-4/IL-13 and LTC_4_ from human basophils (134, 211), indicating that these cells contribute to this allergic disorder.

Early studies reported that up to 80% of food-allergic children exhibit high spontaneous basophil histamine release (212). Moreover, food-allergic children release histamine in response to an IgE-dependent histamine-releasing factor (213). Schroeder and collaborators demonstrated that basophils from food-allergic children also spontaneously release IL-4 and overexpress CD203c (214). Interestingly, spontaneous basophil histamine release and IL-4 secretion decreased in children undergoing sublingual immunotherapy (215). *In vitro* studies show that this enhanced releasability of histamine and IL-4 from basophils of food-allergic children is transferred to basophils of normal subjects by sensitizing normal cells with plasma from the former group. However, the addition of omalizumab during this passive sensitization completely abated the responses, thus pointing to the involvement of IgE in transferring hyperresponsiveness (214).

**Figure 1:**
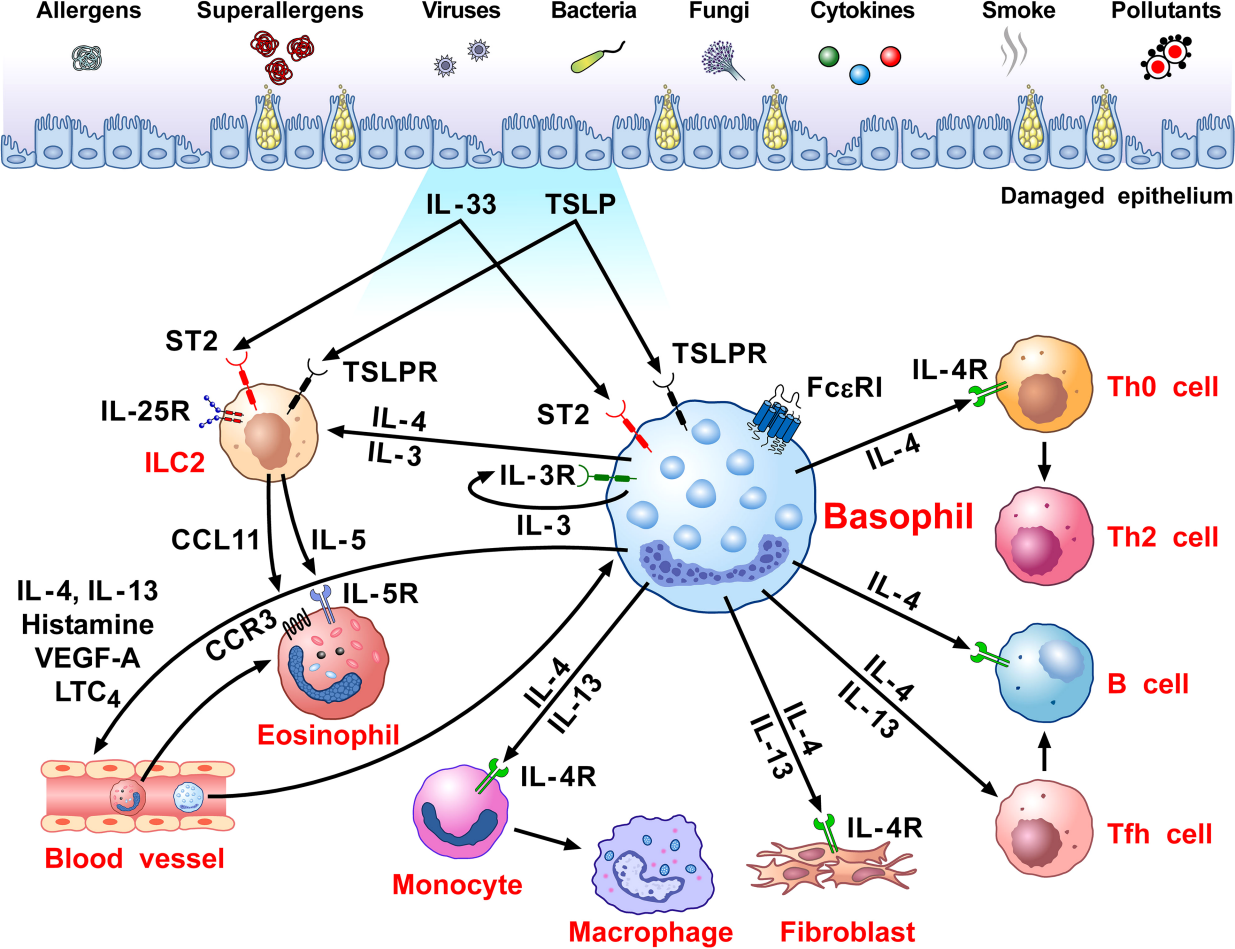
Schematic representation of the versatile role of basophils in the pathobiology of allergic disorders. Several immunological (i.e., allergens, superallergens, viral, bacterial and fungal proteins, cytokines) and non-immunological stimuli (e.g., pollutants, diesel exhaust particles) activate mucosal (i.e., lung and gut) and skin barriers to release different alarmins (i.e., TSLP, IL-33, IL-25) (130, 216, 217). Alarmins activate group 2 innate lymphoid cells (ILC2s) through the engagement of specific receptors (TSLPR, ST2, IL-25R, respectively) (218, 219) to release IL-5 and CCL11 that promote eosinophil infiltration into inflamed tissue (220, 221). Human and mouse basophils express the high-affinity receptor for IgE (FcεRI) (36, 37) and the receptors for IL-3 (IL-3Rα/CD123) (34, 44), GM-CSF (CD116) (45, 46), IL-33 (ST2/IL1RL1) (47–50), IL-5 (CD125) (51) and a variety of chemokine receptors (5, 55–60). The TSLP receptor (TSLPR/IL-7Rα) is expressed by mouse basophils (12, 34), but its presence on basophils from allergic and healthy donors remains controversial (12, 14–16). TSLP activates mouse but not human basophils (12, 17). IL-3 plays a key role in the development, survival and activation of human and mouse basophils (17). IL-3 activates human and mouse basophils to release cytokines and chemokines (12, 17, 46, 222). IgE-FcεRI crosslinking by antigens, superallergens and functional anti-IgE autoantibodies activates basophils to release a wide spectrum of inflammatory and immunomodulatory factors (24, 70, 75, 76, 78, 79, 134, 211, 223). IL-33 activates human and mouse basophils to release several cytokines and chemokines (12). Activated human (12, 24, 74–82) and mouse (12, 47, 80) basophils secrete large amounts of IL-4. Both human (12, 75, 76, 78, 81–85) and mouse basophils (12, 47) also release IL-13. Human basophils secrete several angiogenic factors such as vascular endothelial growth factor-A (VEGF-A) (64). Basophil-derived IL-4 activates ILC2s to enhance the release of IL-5 and CCL11, leading to eosinophil infiltration (68). IL-4 promotes Th2 cell differentiation and enhances humoral immune responses (224). IL-4, together with IL-13, induces T follicular helper cells (Tfh) to promote IgE responses (225, 226). Basophil-derived IL-4 and IL-13 act on inflammatory monocytes inducing their differentiation into M2 macrophages (227). IL-4 and IL-13 activate fibroblasts to promote the production of chemokines (CCL5 and CCL11) (228) and collagen (229). IL-4 and IL-13 and vasoactive mediators (histamine, LTC_4_, VEGF-A) act on blood endothelial cells (64, 230) to upregulate the expression of vascular cell adhesion molecule-1 (VCAM-1) (231), leading to enhanced trans-endothelial migration of eosinophils and basophils (186).

**Figure 1** schematically illustrates the versatile contribution of basophils and their mediators to the development of allergic disorders.

### Peripheral blood basophils in human hematological tumors

Polycythemia vera (PV) is a clonal proliferation of erythroid, megakaryocytic, and myeloid cell lines (232, 233). More than 90% of patients with JAK2-STAT activating mutations (JAK2V617F or exon 12 mutations) are characterized by an overactive JAK-STAT pathway (234, 235). Pruritus and increased basophil-derived mediators (e.g., histamine) are common in PV patients (233, 236, 237). Peripheral blood basophils (238) and CD63 expression are increased in PV patients and are hyperresponsive to IL-3. Increased releasability of histamine from PV basophils can contribute to pruritus in these patients.

Basophilia can develop during the advanced phase of chronic myeloid leukemia (CML) (239) and the transcription factor IKAROS is reduced in the bone marrow from these patients (240). Basophils from CML patients express HGF, promoting CML cell expansion (91). In a mouse model of CML, basophil-derived CCL3 promotes CML development (241, 242). The presence of basophilia is considered an independent risk factor for the progression from myelodysplastic syndrome to acute myeloid leukemia (243, 244).

### Basophils in solid cancers

Basophils are physiologically present in low numbers in peripheral blood. Under certain inflammatory circumstances, the number of circulating basophils can be altered, activated, or migrate from the bloodstream to the sites of inflammation (23, 245). Increased and decreased peripheral blood basophils can be associated with the progression of certain human solid cancers (**Table 1**) (256, 257). Basophilia positively correlates with improved outcomes in melanoma (246, 247), ovarian cancer (248), non-small cell lung cancer (NSCLC) (251), and glioblastoma (252), while basopenia is associated with a poor prognosis for colorectal cancer (245, 249, 250). Basophilia is also linked to improved outcomes in melanoma patients receiving immunotherapy (247). By contrast, in other solid tumors, such as prostate (253) and gastric cancers (250), a detrimental role of circulating or tissue-infiltrating basophils has been reported. Moreover, baseline basophil count predicts recurrence in bladder cancer patients receiving bacillus Calmette-Guérin (BCG) following resection (254). Interestingly, in a mouse model of breast cancer, basopenia correlated with an increased number of pulmonary metastasis (258). However, basophils are not associated with prognosis in breast cancer patients (259). Basophils may support humoral immunity by secreting several B-cell modulating molecules. Once activated, basophils may express CD40L, IL-4, and IL-6 to sustain B-cell proliferation and empower the production of IgM and IgG1. Gomez and colleagues demonstrated that, *in vitro* and *in vivo*, basophils sustain plasma cell survival (245, 260). Histamine is released from basophils and it has been suggested that it can be involved in colon carcinoma (CRC) (245).

**Table 1:** Role of peripheral blood basophils in human solid cancers.

Tumor type	Prognostic/predictive role	Reported observation	References
**Melanoma**	Favourable	Basophilia is associated with improved outcome in melanoma patients receiving immunotherapy with nivolumab plus ipilimumab and in newly diagnosed stage I-II melanoma patients	(246, 247)
**Ovarian cancer**	Favourable	A higher frequency of circulating basophils and the presence of activated basophil signature are associated with improved overall survival in ovarian cancer patients	(248)
**Colorectal cancer**	Favourable	Low pretreatment basophil counts are associated with worse prognosis and higher tumor aggressiveness in colorectal cancer patients	(245, 249, 250)
**NSCLC**	Favourable	Higher basophil counts are associated with increased probability of responding to ICI therapy in two cohorts of stage-IV NSCLC patients	(251)
**Glioblastoma**	Favourable	Increased pre-operation circulating basophils predict better progression free survival in patients	(252)
**Prostate cancer**	Unfavourable	Elevated baseline basophils and basophil-to-lymphocyte ratio are associated with worse clinical outcomes in metastatic hormone sensitive prostate cancer patients	(253)
**Bladder cancer**	Unfavourable	Baseline basophil count may predict recurrence in BCG-treated primary bladder cancer patients	(254)
**Gastric cancer**	Unfavourable	Elevated baseline basophil counts are prognostic for unfavorable clinical outcomes in gastric cancer patients treated with ICI plus chemotherapy,	(255)

BCG, Bacillus Calmette-Guérin; ICI, immune checkpoint inhibitors; NSCLC, non-small cell lung cancer.

Bax and coworkers have investigated the presence and functions of basophils from peripheral blood and in ovarian cancer (248). The same group reported that basophilia and basophils who possess greater ability for *ex vivo* stimulation are associated with improved outcomes (261). Additionally, a positive correlation between improved progression-free survival of patients and activated basophil markers (CD63^+^, CD203c^+^, CD123, CCR3, FcεRI) was observed in the TME of ovarian cancer (Bax, Chauhan et al., 2020). These results indicate that activated peripheral blood and intratumoral basophils correlate with a survival benefit in ovarian cancer patients (261). Nevertheless, these favorable effects that basophils might mediate in targeting the tumor for destruction can potentially result in unfavorable outcomes. For example, basophils have been found in ascitic fluid from ovarian cancer patients and it has been suggested that their release of vasoactive mediators (e.g., histamine) may exacerbate fluid accumulation in the peritoneal cavity (58).

It has been reported that the expression of cytokines by lung-resident basophils can be induced by local signals (e.g., IL-33, GM-CSF) (47, 102), emphasizing the plasticity of these cells. Hence, the lung microenvironment might influence the transcriptional and functional development of basophils. Likewise, these resident basophils seemingly play an important role in lung development and function by forming cellular networks and facilitating so-called macrophage imprinting. Low percentages of basophils (0.4%) were located in the immune infiltrate of human non-small cell lung cancer (NSCLC) tumors (262). Basophils have been identified in the immune landscape in early (stage I) lung adenocarcinoma and in non-involved lung tissue (nLung) (102). It remains unclear what the exact function basophils mediate in the TME, yet emerging evidence points to their capacity to secrete IL-4 and IL-13 as playing a potential role. For example, mouse and human studies have shown that basophils, by secreting these cytokines, facilitate the development/expansion of M2-like monocytes/macrophages (227, 263–265), which are often a prominent part of the immune cell landscape of the TME. However, in chronic inflammation, the exposure of basophils to certain cytokines, such as IL-33, may induce the polarization of lung macrophages to M2-like phenotype characterized by the expression of anti-inflammatory genes *Clec7a, Arg1, Itgax*. In this context, basophils are participants in the inflammatory entourage in lung cancer (261). It seems equally possible that basophil-derived IL-4/IL-13 also favor tumorigenesis by diminishing Th1-like immunity that is better suited to contest the cancer (80). Should these hypotheses prove correct, then another important question that arises pertains to the endogenous stimulus responsible for inducing these cytokines. In this regard, Schroeder et al. demonstrated that purified human basophils release histamine, IL-4 and IL-13 when co-cultured with the lung adenocarcinoma cell line A549 (16). Unexpectedly, these effects required IgE-expressing basophils and were suppressed by specific inhibitors of FcεRI signaling. A subsequent study revealed that the IgE-binding lectin, galectin-3, expressed on the A549 cells, was responsible for this model of basophil activation (223). In fact, galectin-3 is a biomarker and/or factor implicated in many kinds of cancer, chronic inflammation, cardiovascular disease, autoimmunity, and also beneficially in wound healing (266). These results thus reveal an innovative mechanism by which galectin-3 expressed by human lung carcinoma cells are able to activate basophils [and likely other cell types, namely dendritic cells (DCs) and monocytes] (267) to release cytokines and pro-inflammatory mediators. Further studies are necessary to understand the role of galectin-3 in activating basophils, and how IL-4/IL-13 and other mediators could contribute to human and experimental lung cancer.

Interestingly, many immune cells and markers that have a mounting prominence in cancer/tumorigenesis are also observed in experimental models of wound healing. For example, scaffolds that promote wound-healing often induce Th2 immune responses, whereby IL-4 and IL-13 are recognized as critical cytokines that help initiate the process (268). M2 cells, whose development is often dependent on the actions of IL-4/IL-13, are also widely implicated in wound healing. Not surprisingly, much emphasis is placed on the role of Th2 cells in being the source of IL-4/IL-13. However, in a recent publication that explored the mechanisms associated with wound healing following experimental myocardial infarction (MI), basophils were identified as a critical source of IL-4/IL-13 required for the healing process. Specifically targeting basophils using conditional knockouts or by antibody-mediated depletion, significantly impaired this wound healing (269). Moreover, the administration of IPSE-α1, an IgE-binding glycoprotein isolated from helminth eggs and well known for activating basophils for IL-4/IL-13, greatly augmented healing following the MI. While the endogenous ligand for stimulating IL-4/IL-13 from basophils in this model was not reported, it is intriguing to speculate that galectin-3 is involved. Indeed, galectin-3 is often a prominent marker in wound healing, both at the transcriptional and protein levels (266).

Investigation on the role of basophils in models of melanoma has provided interesting results in Foxp3^DTR^ mice, in which these cells caused melanoma rejection (270). CCL3 and CCL4 produced by intratumoral basophils induced CD8^+^ lymphocyte recruitment in TME. The administration of FcϵRI (MAR-1) mAb in Foxp3^DTR^ melanoma-bearing mice depleted basophils and abrogated the recruitment of CD8^+^ T cells thus preventing the rejection of melanoma. Furthermore, the IL-3/anti-IL-3 antibody complexes combined with adoptive T cell transfer induced basophilia and consequent T cell infiltration, which positively correlated with melanoma rejection. Unfortunately, the MAR-1 antibody can also deplete/activate other immune cells (e.g., mast cells, DCs, monocytes) which express FcϵRI (271, 272). Thus, studies in newer genetically engineered basophil-deficient mouse models (80, 97) appear necessary to establish the role of basophils in melanoma.

IL-33 is a cytokine that induces tumoricidal functions in eosinophils (273, 274) and upregulates granzyme B mRNA and the surface expression of CD63 (67), suggesting phenotypic and functional activation. Moreover, IL-33-activated basophils co-cultured with B16.F10 melanoma cells, inhibited tumor cell-growth compared to melanoma cells co-cultured with unstimulated basophils (67).

In a pioneering observation, Ann M. Dvorak first demonstrated piecemeal degranulation of basophils in human pancreatic cancer (PC) (7). Elegant studies evaluated the role of basophils in experimental and human ductal adenocarcinoma (PDAC) (80). In PDAC patients, they identified *IL4* expressing basophils in tumor-draining lymph nodes (TDLNs). Basophils in TDLNs were an independent negative prognostic biomarker of patient survival. They also evaluated basophil role in PC using the *Mcpt8*-Cre basophil deficient (275) and wild-type (WT) mice. After PC implant, cancer was detected in 80% WT, but not in basophil-deficient mice. Basophils were found in TDLNs and cancer-associated fibroblasts (CAFs) released TSLP, which activated DCs to produce IL-3 from CD4^+^ T cells. CCL7, produced by DCs and CD14^+^ monocytes, induced basophil migration into TDLNs. Basophils activated by IL-3 played a pro-tumorigenic role through the production of IL-4, which favored Th2 and M2 polarization. These findings are consistent with our results indicating that basophil-derived IL-4 (and IL-13) promote M2-like cells (263).

Topical exposure of the skin of mice to an environmental DNA-damaging xenobiotic [i.e., 7,12-dimethylbenz [a] anthracene (DMBA)] caused the development of squamous-cell carcinomas (SCCs), high serum levels of IgE and tumor infiltration of IgE-bearing basophils (276). In this model, FcϵRI^+^ basophils mediated the DMBA-induced IgE protection against carcinogenesis. In contrast, topical exposure of the skin of mice to the proinflammatory agent 12-0-tetradecanoylphorbol-13-acetate (TPA) increased serum IgE and IgE-bearing basophils in the skin that promoted carcinogenesis (97). In a two-stage model of epithelial carcinogenesis (DMBA and subsequent exposure to TPA), Hayes and coworkers also discovered that mice lacking IgE (*lgh7^-/-^*) were less responsive to tumor development compared to WT mice (97). IgE-signaling was crucial for mediator release from basophils and infiltrating tissue basophils showed expression of *Cxcr2, Cxcr4*, and *Ptgdr2* (CRTH2, the PGD_2_ receptor). Basophil infiltration into the inflamed skin was mediated by TSLP/IL-3-mediated upregulation of CXCR4 on basophils. The *Mcpt8* ^Cre/+^ mice, presenting normal mast cell numbers but strongly reduced basophils (275), were less responsive to tumor growth. **Table 2** summarizes the role of basophils in the TME of different solid cancers.

**Table 2:** Role of basophils in tumor microenvironment.

Tumor type	Effect on cancer	Observed role	Mechanism	References
**Melanoma**	Anti-tumoral	Treg depletion results in infiltration of basophils and CD8^+^ T cells in the TME that promote tumor rejection in mice IL-33-activated mouse basophils induce melanoma cell death *in vitro*	CCL3/CCL4 secretion by intratumoral basophils induces CD8^+^ T cell recruitment in TME Release of Granzyme-B	(270) (67)
**Ovarian cancer**	Anti-tumoral	Activated signature (CD123, CCR3, FcϵRI, CD63, CD203c gene expression) in tumor-resident basophils is associated with improved outcomes in these patients	NA	(248, 261)
**Lung cancer**	Anti-tumoral	Higher expression of basophil markers (CD123, CCR3, and FcϵRI) in tumors is associated with improved overall survival in lung cancer patients	NA	(261)
	Pro-tumoral	Lung inflammatory cytokines trigger basophil-induced M2 polarization	Basophil secretion of IL-4/IL-13	(47)
**Skin cancer**	Anti-tumoral	Topic exposure to DNA-damaging carcinogen DMBA promotes tumor-protective IgE response through skin infiltrating basophils	Possible release of cytotoxic soluble mediators	(276)
	Pro-tumoral	Skin inflammation by TPA, MC903 or R848 induced IgE/FcϵRI-signalling in basophils promote epithelial carcinogenesis	TSLP/IL-3-mediated upregulation of CXCR4 on basophils	(97)
**Gastric cancer**	Pro-tumoral	Increased tumor-infiltrating basophils in tissues from gastric cancer patients are negatively associated with therapy response	Increased tumor M2 macrophage infiltration	(255, 277)
**Pancreatic cancer**	Pro-tumoral	IL-4-secreting basophils are significantly increased in TDLNs of PDAC patients, correlate with predominant Th2 inflammation and represent an independent prognostic factor of poorer survival after surgery	Recruitment in TDLN mediated by alternatively activated monocyte-secreted CCL7/MCP3	(80)

DMBA, 7,12-dimethylbenz [a] anthracene; MC903, vitamin D3 analogue; NA, not assessed; PDAC, pancreatic ductal adenocarcinoma;R848, resiquimod; TPA, 12-O-tetradecanoylphorbol-13-acetate.

Colony-stimulating factor 1 (CSF1) is a primary regulator of monocytes/macrophage that sustains macrophage polarization towards an M2-like phenotype (278). Mouse basophils resident in the lung express high levels of *Csf1* and contribute to M2 polarization of lung macrophage (47). The functional relevance of basophil-derived CSF1 was also underlined *in vivo* in a murine model of atopic dermatitis, where it promoted M2-like macrophage polarization (279). Interestingly, an inhibitor of CSF1/CSF1 receptor signaling reduced tumor-associated macrophage (TAM) infiltration in the TME of sarcoma models (278). These experimental findings may have translational relevance in cancer: there is the possibility that CSF1, in conjunction with basophil-derived IL-4/IL-13, might enhance the M2-like/TAM polarization of macrophage in TME (280).

**Figure 2:**
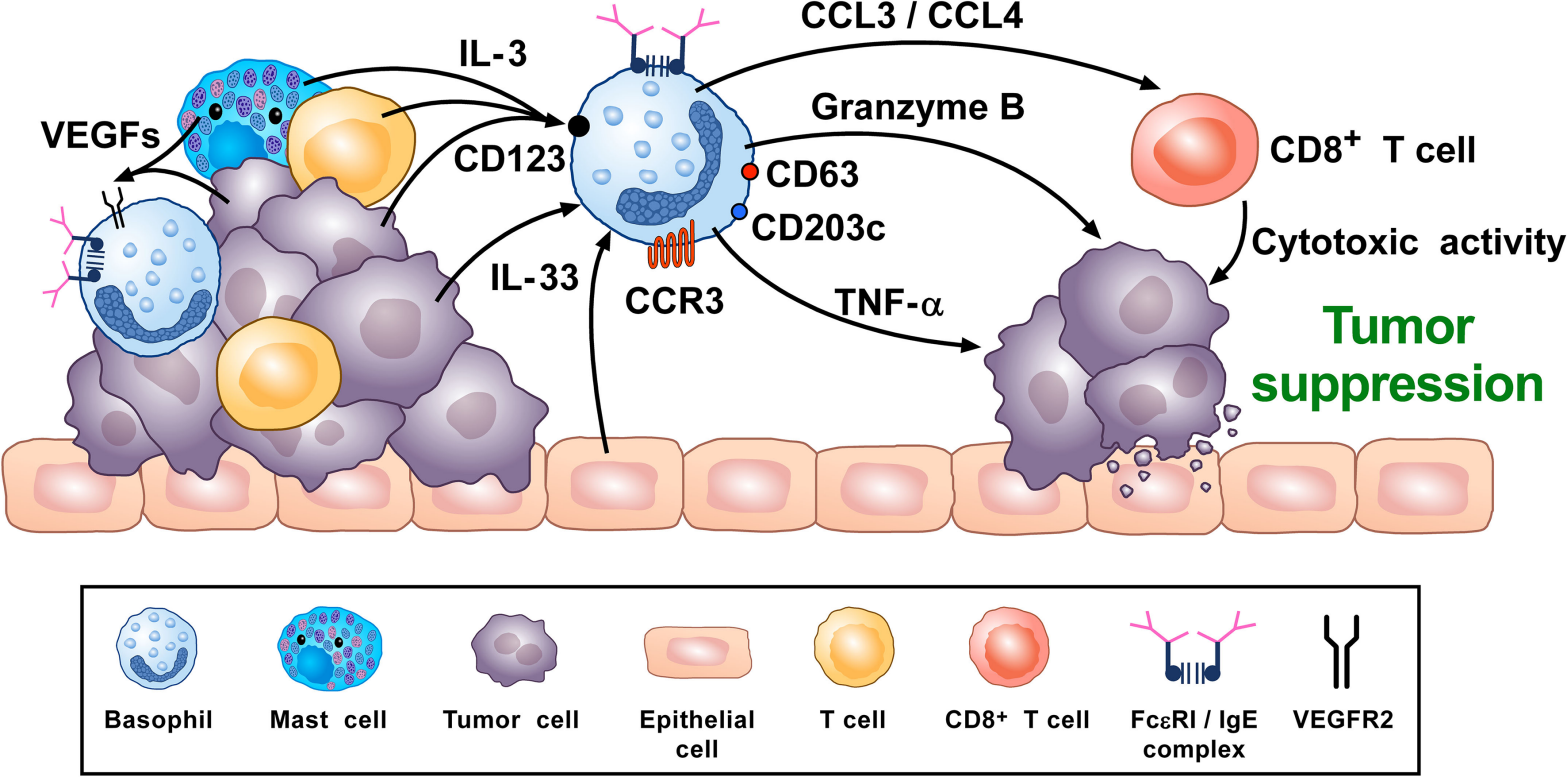
Theoretical representation of how basophils can promote tumor suppression. Basophils have been located in the immune infiltrate of several human (80, 102, 261, 262) and experimental tumors (80, 97, 102, 276). Vascular endothelial growth factors released by cancer cells and immune cells in tumor microenvironment (TME) (e.g., mast cells, macrophages) (118, 120–123) can favor basophil infiltration in TME through the engagement of VEGFR2 on these cells (120). IL-3, produced by intratumoral lymphocytes, mast cells and cancer cells (17, 82, 281), is the most important growth and activating factor for human and mouse basophils, through the engagement of the IL-3 receptor (IL-3Rα/CD123) (17). CCL3/CCL4 secreted by intratumoral basophils induces CD8^+^ T cell recruitment in TME, promoting melanoma rejection in mice (270). IL-33, a pleiotropic cytokine produced by epithelial and tumor cells (282), plays a central role in tumorigenesis (282). IL-33 upregulates granzyme B mRNA and the surface expression of CD63, suggesting functional and phenotypic basophil activation. IL-33-activated mouse basophils induce melanoma cell death *in vitro* (67). Mouse (47, 86) and, under specific circumstances, human basophils (88, 89) release TNF-α. Human and mouse basophils release granzyme B (66, 67). Both TNF-α and granzyme B exert cytotoxic effects on tumor cells (68, 69). Activated signature (CD123, CCR3, CD63, CD203c gene expression) in tumor resident basophils is associated with improved outcome in ovarian cancer patients (248, 261). Topical exposure to a DNA-damaging carcinogen promotes tumor-protective IgE response through skin infiltrating basophils (276). Taken together, these results suggest that, in certain experimental and clinical conditions, basophils and their mediators may play an anti-tumorigenic role.

**Figure 3:**
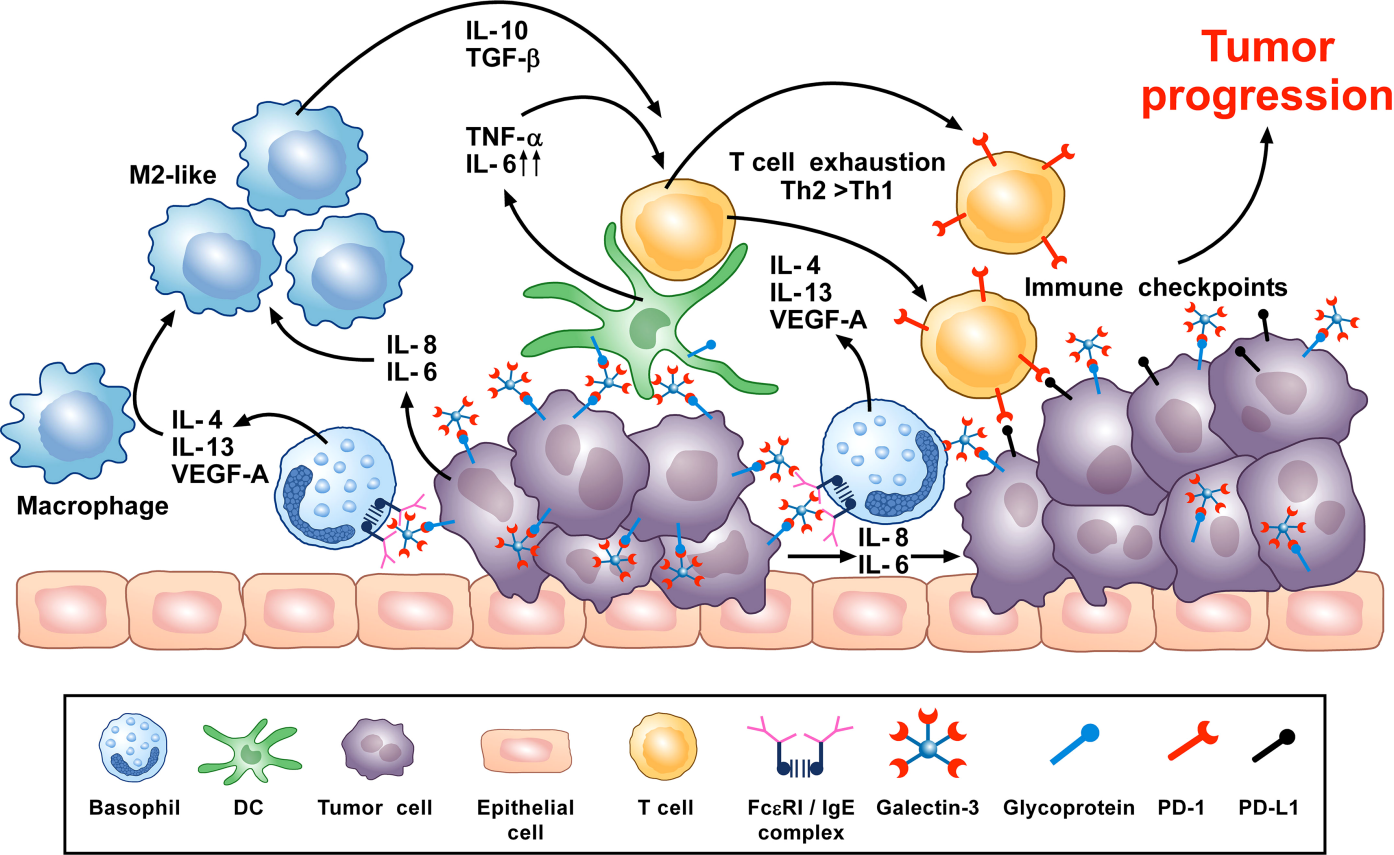
Theoretical representation of how basophils can promote tumor progression. Galectin-3 (Gal-3) is a lectin highly expressed by many types of cancer cells, frequently manifesting as a marker of poor prognosis with capacity to mediate immunosuppression within the tumor microenvironment (TME) (266). Recent *in vitro* studies show that Gal-3, expressed by the A549 adenocarcinoma cell line (or EC-Gal-3), has the capacity to activate basophils to secrete copious amounts of IL-4/IL-13 (16, 223). Both cytokines are known to promote M2-like macrophages, which are major players in the TME (227, 263–265). IL-4-producing basophils have been identified in the TME of human pancreatic cancer, with mouse models indicating that this IL-4 promotes a Th2>Th1 response that is more conducive to tumorigenesis (80). Additionally, basophils are long known to secrete VEGF-A (64) that promotes angiogenesis. Other studies show that basophils can induce IL-6/IL-8 secretion from cell lines through a mechanism requiring cell-to-cell contact (283) (JTS, unpublished). This tumor cell-derived IL-6/IL-8 is implicated in playing a critical role in metastasis formation (284). Likewise, dendritic cells and monocytes activated by EC-Gal-3 are shown to produce high levels of TNF-α/IL-6 *in vitro* (285). Chronic production of these cytokines, when combined with M2 cell-derived IL-10/TGF-β, are implicated in promoting T-cell exhaustion by up-regulating checkpoint inhibitors (e.g., PD-1) that interact with tumor cell-associated markers (PD-L1) to suppress cytotoxic T cell activity (286). V-domain immunoglobulin suppressor of T-cell activation (VISTA) is another immune checkpoint receptor which plays a role in cancer progression (287, 288) and regulates allergen-specific Th2-mediated immune responses (289). Overall, it is proposed that the combined actions of these dysregulated innate immune responses synergize to promote tumorigenesis.

A synopsis of the above findings signifies some conflicting views of the role that basophils potentially mediate in tumorigenesis. A more classical interpretation (from the ovarian, lung, colorectal, and melanoma data) suggests basophils mediate anti-tumor effects (248, 261, 270, 276). While the mechanisms underlying the beneficial outcomes are poorly defined, it has been proposed that some basophil-derived mediators (e.g., granzyme B and TNF-α) exert tumoricidal activity while others (e.g., CCL3 and CCL4) facilitate the recruitment of cytotoxic CD8^+^ T cells (**Figure 2**). In contrast, there is growing evidence that basophils, under certain circumstances, can promote tumorigenesis (**Figure 3**). In this instance, the tumor cell itself seemingly modulates basophil responses, causing a release of cytokines that favor the development of protumorigenic TME. Interestingly, this latter scenario shares many similarities with that seen in wound healing.

## Conclusions

Basophils were initially considered as effector cells of allergic diseases (166, 230). The discovery that murine (290) and human basophils produce immunomodulatory cytokines (e.g., IL-4, IL-3, and IL-13) (28, 76–78, 81, 85, 291) changed dramatically this erroneous concept. In addition, human and murine basophils release several canonical (24, 64, 90, 91) and non-canonical angiogenic factors (133) that play a pivotal role in inflammatory and tumor angiogenesis. Further *in vitro* and *in vivo* studies are needed to investigate the contribution of angiogenic factors released by mouse and human basophils in experimental and human tumors.

Basophils have been identified in human lung (102), gastric (99, 100), pancreatic (7, 80) and ovarian cancer (248). Lung-resident basophils (47) can provoke M2 polarization of lung macrophages, as occurs in several tumors (105, 106). The presence of basophils and their activation signatures appear to be linked with more favorable patient outcomes in certain tumors (melanoma, lung cancer, ovarian cancer) (248, 261, 270). Otherwise, with particular reference to gastric and pancreatic cancers, increased tumor-infiltrating basophils are negatively associated with less favorable overall survival (80, 255, 277).

Basophil functions *in vivo* have been evaluated through several models of basophil-deficient mice (275, 292–294). It should be remembered that, in some instances, studies using antibody-depleted basophils have produced erroneous findings due to lack of antibody specificity (271, 272) and even new mouse basophil-targeted mutants have some off-target hematological alterations (295). Therefore, the evaluation of basophil functions in complex and heterogeneous disorders, such as cancer and allergic diseases using multiple genetically engineered models of basophil deficiency, demands caution in data interpretation.

Collectively, recent findings highlight the critical contributions of basophils during homeostatic conditions and beyond their ability to promote allergic inflammation. Further studies are needed to understand the mechanisms and environmental factors driving basophils to play a pro- or anti-tumorigenic role in experimental and human cancers. A better knowledge of the involvement and functions of basophils in human immunity appears necessary considering the participation of these cells in immune and cancer cell crosstalk and in priming of several immune cell types. Single-cell RNA-seq of the immune landscape of tumor cells will be of paramount importance to characterize the role of basophils in different types of human and experimental cancer. Understanding of the molecular mechanisms orchestrated by basophils in the TME of several cancer types could allow to develop novel pharmacological/immunological strategies to modulate basophil functions and perhaps to prevent tumor progression.

## Author contributions

RP and ARG are co-first authors of this manuscript. All authors contributed to the article and approved the submitted version. GS and GV are co-senior authors of this manuscript.
